# Early and sustained increase in time in range 1 year after initiation of hybrid close loop therapy via telemedicine in type 1 diabetes patients

**DOI:** 10.1007/s00592-023-02051-w

**Published:** 2023-04-03

**Authors:** Ana M. Gómez, Diana Henao, Darío Parra, Alfonso Kerguelen, Pablo Jaramillo, Yaline Gómez, Oscar Mauricio Muñoz, Martin Rondón

**Affiliations:** 1 Hospital Universitario San Ignacio, Pontificia Universidad Javeriana, Carrera 7 No. 40-62, Bogotá, Colombia; 2 Hospital Universitario San Ignacio, Endocrinology Unit, Bogotá, Colombia; 3 Department of Internal Medicine, Hospital Universitario San Ignacio, Bogotá, Colombia; 4 Department of Clinical Epidemiology and Biostatistics, Pontificia Universidad Javeriana, Bogotá, Colombia

**Keywords:** Time in range, Hybrid closed loop (HCL), Sensor-augmented pump therapy (SAPT), Automated insulin delivery system (AID), Type 1 diabetes, Telemedicine

## Abstract

**Background and Aims:**

Evidence supports the efficacy and safety of the Hybrid Close loop (HCL) system in patients with type 1 diabetes (T1D). However, limited data are available on the long-term outcomes of patients on HCL with telemedicine follow-up.

**Methods:**

A prospective observational cohort study including T1D patients, who were upgrading to HCL system. Virtual training and follow-up were done through telemedicine. CGM data were analyzed to compare the baseline time in range (TIR), time below range (TBR), glycemic variability and auto mode (AM), with measurements performed at 3, 6 and 12 months.

**Results:**

134 patients were included with baseline A1c 7.6% ± 1.1. 40.5% had a severe hypoglycemia event in the last year. Baseline TIR, measured two weeks after starting AM was 78.6 ± 9.94%. No changes were evident at three (Mean difference − 0.15;CI-2.47,2.17;*p* = 0.96), six (MD-1.09;CI-3.42,1.24;*p* = 0.12) and 12 months (MD-1.30;CI-3.64,1.04;*p* = 0.08). No significant changes were found in TBR or glycemic variability throughout the follow-up. Use of AM was 85.6 ± 17.5% and percentage of use of sensor was 88.75 ± 9.5% at 12 months. No severe hypoglycemic (SH) events were reported.

**Conclusions:**

HCL systems allow to improve TIR, TBR and glycemic variability safely, early and sustained up to 1 year of follow-up in patients with T1D and high risk of hypoglycemia followed through telemedicine.

## Introduction

Achieving glycemic control in patients with type 1 diabetes (T1D) is challenging. The T1D Exchange Registry showed that only 21% of adults meet A1c goals [1]. Even though we currently have different tools such as insulin analogs, continuous glucose monitoring system and insulin infusers however, less than 10% of patients achieve the HbA1c < 6.5% goals and only 20% reach the 7.0% goal [2]. Additionally, hypoglycemia persists as a limiting factor for optimal glycemic control. The relationship between hypoglycemia and increased global and cardiovascular mortality, and morbidity has been described [3].

The hybrid closed loop (HCL) systems integrate continuous glucose monitoring with an insulin infuser and an algorithm that automatically adjusts the basal insulin infusion, allowing the use of boluses of insulin at each meal by the user. This therapy has shown benefits in reducing HbA1c, hyperglycemia, hypoglycemia and glycemic variability, and increasing the time in the range between 70 and 180 mg/dl, in patients with DM1 older than 7 years [4, 5]. However, these data comes from short follow-up pivotal studies.

Since 2020, and specially related with COVID 19 pandemic, the use of telemedicine for the education and follow-up of patients with this technology has increased, demonstrating that the benefits of therapy were maintained [6]. However, there is little data on long-term follow-up through telemedicine in real life. The objective of this study is to describe the efficacy and safety of the HCL system up to one year of follow-up through telemedicine.

## Methods

A prospective observational cohort study was conducted, including patients with T1D who were upgrading to HCL system (Minimed 670G insulin pump, Medtronic, Northridge, CA, USA) at Hospital Universitario San Ignacio in Bogotá, Colombia. Recruitment was performed between March 2020 and January 2021. T1D patients older than 14-years-old and treated with multiples doses of insulin (MDI), Sensor Augmented Pump (SAP) Therapy with Low Glucose Suspend (LGS) (Paradigm VEO®, Medtronic MiniMed, Inc, Northridge, CA, USA) or Sensor Augmented Pump Therapy with Predictive low-glucose management (SAP-PLGM) (MiniMed 640G®, Medtronic MiniMed, Inc, Northridge, CA, USA) were invited to participate. Patients with alcohol consumption, those who refused to sign the informed consent and pregnant women were excluded. The Ethics Committee of Hospital Universitario San Ignacio and Pontificia Universidad Javeriana approved the study.

The virtual training was directed by a diabetes expert physician, and was supported by the education and nutrition teams. The protocol was described in a previous publication [6]. Briefly, all patients received three sessions of training about the use of device in manual and auto mode, and the way to upload CGM data to Carelink ™. Patients with MDI and Paradigm VEO® as baseline therapy had two additional sessions, including carbohydrate counting, device overview and basic concepts about continuous glucose monitoring. All virtual sessions were performed through Zoom Enterprise Version of the Zoom video conferencing application (Zoom Video Communications, San Jose, California). The device was programed according Medtronic clinical recommendations [6]. Subjects treated with SAP-LGS or SAP-PGLM therapy and time in range (70–180 mg/dl) above 70% continued with their baseline settings. The PLGM function was indicated to be turned on with a threshold of 60 mg/dl. For patients with history of severe hypoglycemia (SH) and hypoglycemia unawareness (HU), a threshold of 70 mg/dl was set. Active insulin function was set to three hours, except if the glomerular filtration rate (GFR) was below 30 ml/min.

The basal TIR was recorded after two weeks of automatic mode. Follow-up was carried out through telemedicine every month. Baseline data, including HbA1c (high-efficiency liquid chromatography), creatinine and albuminuria/ creatinuria ratio was collected through virtual consultation. The Clarke questionnaire was carried out at baseline, 3 and 12 months, and serious adverse events, such as SH, diabetic ketoacidosis, hospitalization, infection at the cannula insertion site, or device dysfunction were recorded at each virtual session. CGM data was uploaded by the patients using a virtual platform (CareLink Pro® version 4.0 C, Medtronic MiniMed, Inc, Northridge, CA, USA) at 3, 6, 9 and 12 months. At each of these moments, the CGM information for the last 2 weeks were downloaded and imported into a MATLAB® calculation software for analysis.

SH was defined as the need for assistance from a third person for recovery and HU was detected using the Clarke questionnaire with a score ≥ 4 [7]. HCL discontinuers were defined as participants who had < 10% of auto mode use at any visit [8]. A multidisciplinary group (MT) was defined as a group composed by a diabetes specialist, with the support of a diabetes nurse educator and nutritionist who follows-up the patient. Usual care (UC), was defined as medical follow-up according to the insurer's protocols without this support.

**Fig. 1:**
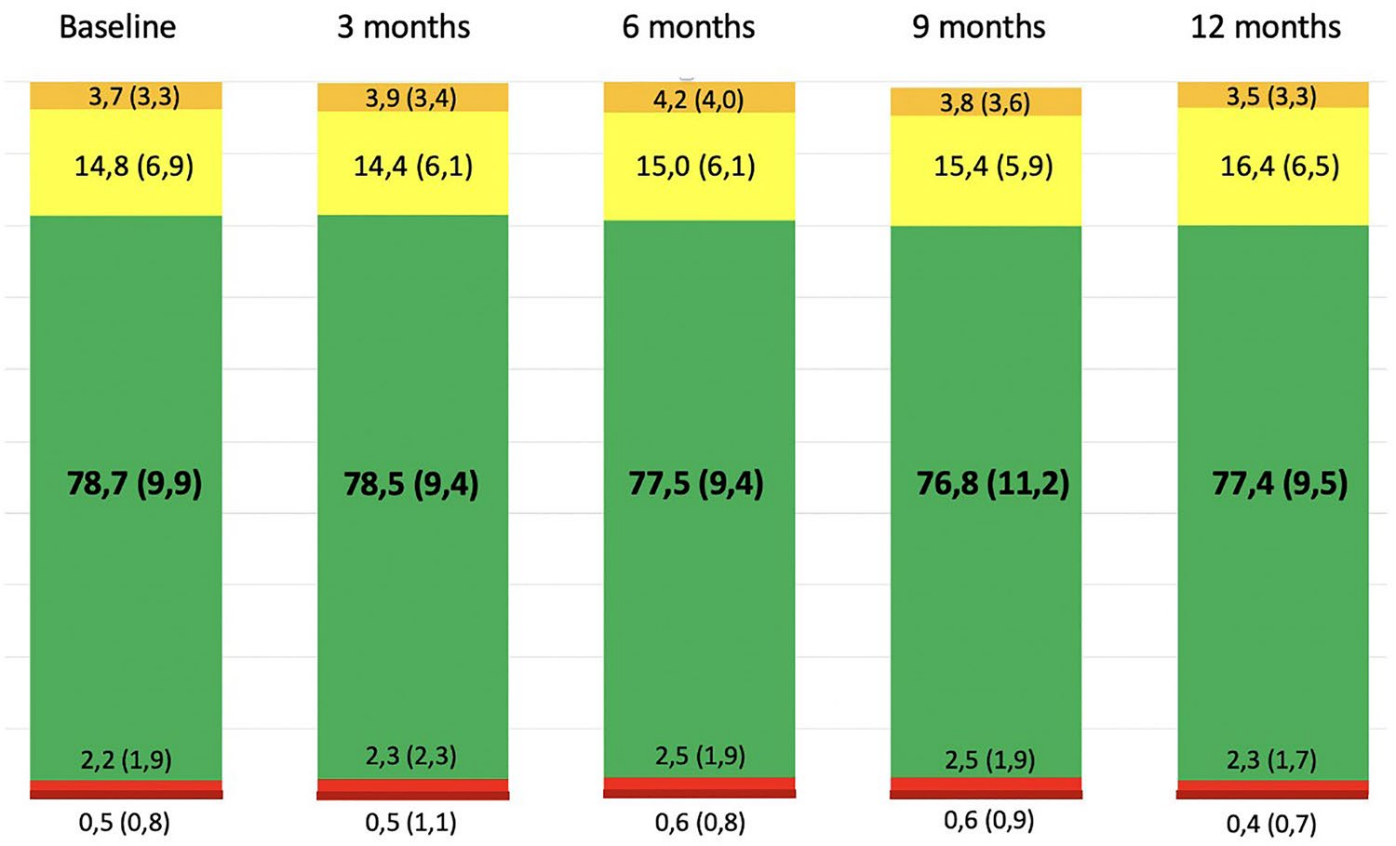
Internationally recongnized and clinically important time in ranges (bars). The figures shows the early and sustained increase in time in range 1 year after initiation of hybrid close system

For continuous variables, mean and standard deviation (SD) or median and interquartile ranges (IQR) were reported, according with variables characteristics. For categorical variables, frequencies and percentages were reported. CGM data were pre-processed from the records to discard monitoring days with consecutive losses greater than 50 samples, lower losses were linearly interpolated. The data of each patient were organized by calendar days (00:00 to 23:59 h) [9]. Based on these data, we calculated the metrics of time in range (TIR), time below range (TBR) (< 54 y < 70 mg/dL), time above range (TAR) (> 180 mg/dL), and coefficient of variation (CV). Comparisons between basal and follow-up measures were made using paired *t* test considering that these were repeated measurements. Subgroups analysis were performed according to prior therapies and follow-up strategies (Fig. 1). The confidentiality and privacy of the data were protected maintaining the collection formats under secure access. STATA version 16.0 was used for the analysis.

## Results

Baseline demographic and clinical characteristics are shown in Table 1. 134 patients were included in the analysis. 54.9% were women and the average age was 38 ± 13.7 years. Hypoglycemia history was the main indication for upgrade to HCL system (48.8%) with HU detected in 32.4% of patients. About 60% of the patients had been previously managed with sensor augmented therapy. The prevalence of SH before the use of HCL system was 48.9% in patients previously treated with MDI.

**Table 1:** Baseline characteristics of included patients

Baseline characteristics	*n* = 134
Age in years, mean (SD)	38 (13.7)
Female, n (%)	83 (54.9)
Duration of diabetes in years, mean (SD)	18.6 (10.4)
Creatinine, mg/dL, mean (SD)	1.2 (1.5)
Body mass index, mean (SD)	24.4 (3.2)
Baseline A1c, mean (SD)	7.6 (1.1)
*Indication for use insulin pump, n(%)*	
Hypoglycemia	62 (48.8)
Poor metabolic control	30 (23.6)
High glycemic variability	32 (25.2)
Insulin resistance	2 (1.5)
*Basal treatment, n (%)*	
MiniMed® 640G with SmartGuard	22 (16.7)
Paradim VEO	58 (43.9)
MDI	51 (38.6)
*Macrovascular complications n (%)*	
Myocardial infarction	6 (4.5)
*Microvascular complication n (%)*	
Diabetic retinopathy	19 (14.3)
Diabetic nephropathy	17 (12.7)
Diabetic neuropathy	13 (9.8)
Diabetic gastroparesis	6 (4.5)
*Severe hypoglycemia in the last year, n (%)*	49 (40.5)
*Hypoglycemia unawareness, n (%)*	
Clarke questionnaire score ≥ 4	36 (32.4)
*Diabetic Ketoacidosis in the last year, n (%)*	8 (6.5)
*Multidisciplinary team follow-up, n (%)*	94 (69.6)

SD: standard deviation

The most common microvascular complication was diabetic retinopathy (14.3%), followed by diabetic nephropathy (12.7%) (Table 1). Baseline A1c was 7.6% ± 1.1%. Almost 70% of the patients had follow-up in a multidisciplinary team.

### Efficacy and safety

Mean TIR (70–180 mg/dl) at baseline was 78.6 ± 9.9%. No significant changes were evident at three (Mean difference − 0.15; CI − 2.47, 2.17; *p* = 0.96), six (MD − 1.09; CI − 3.42, 1.24; *p* = 0.12) and 12 months (MD − 1.30; CI − 3.64, 1.04; *p* = 0.08) (Table 2). A subgroup analysis was performed according to the follow-up modality and baseline therapy (Table 3). The initial TIR (70—180 mg/dL) was similar in patients followed by a multidisciplinary team (MT) and usual care (UC) (79.3 ± 10.0% vs 76.5 ± 10.4%, *p* = 0.14). After 12 months, the TIR remained without significant changes, compared with basal values, independent of the follow-up program (UC 75.15 ± 10.84%, *p* = 0.12 and MT 78.29 ± 8.87%, *p* = 0.35). The baseline TIR was higher for patients treated with SAP (80.66 ± 8.95 vs 76.93% ± 10.73), and remained without significant changes at the end of follow-up regardless of prior therapy (Table 3).

**Table 2:** CGM data and adherence total population

	Baseline	3 months	*P* value	6 months	*P* value	9 months	*P* value	12 months	*P* value
TIR (70–180 mg/dL)	78.7 (9.9)	78.5 (9.4)	0.96	77.5 (9.4)	0.12	76.8 (11.2)	0.03	77.4 (9.5)	0.08
TAR (> 180 mg/dL)	14.8 (6.9)	14.4 (6.1)	0.95	15.0 (6.1)	0.66	15.4 (5.9)	0.55	16.4 (6.5)	0.22
TBR (< 70 mg/dL)	2.2 (1.9)	2.3 (2.3)	0.22	2.5 (1.9)	0.02	2.5 (1.9)	0.04	2.3 (1.7)	0.25
TBR (< 54 mg/dL)	0.5 (0.8)	0.5 (1.1)	0.51	0.6 (0.8)	0.11	0.6 (0.9)	0.15	0.4 (0.7)	0.52
GMI	6.7 (0.3)	6.8 (1.7)	0.51	6.74 (0.32)	0.34	6.7 (0.2)	0.20	7.3 (5.5)	0.27
Mean glucose	142.7 (13.9)	142.7 (13.2)	0.67	142.5 (15.3)	0.70	151.9 (92.9)	0.27	145.2 (13.1)	0.07
CV	31.4 (6.0)	31.9 (5.7)	0.28	31.8 (5.4)	0.17	31.8 (5.6)	0.22	32.2 (5.5)	0.09
Adherence									
Time of use of sensor	90.3 (7.3)	91.4 (9.1)	0.18	90.7 (10.0)	0.65	90.5 (9.0)	0.81	88.7 (9.5)	0.07
Time of use of automatic mode	80.4 (23.7)	85.9 (22.5)	0.13	88.1 (17.9)	0.02	86.1 (19.4)	0.10	85.6 (17.4)	0.43

Data presented as mean and standard deviation. TIR: time in range; TAR: time above range; TBR: time below range; GMI: glucose management indicator; CV%: coefficient of variation. *p*-value of paired *t* test comparing with baseline values

**Table 3:** CGM data and adherence according with basal therapy and follow up team

Sub		Baseline		3 months		*p* value	6 months		*p* value	9 months		*p* value	12 months		*p* value
*Follow-up team*															
TIR (70–180 mg/dL)	UC	76.51 (10.42)		76.68 (8.53)		0.91	76.80 (10.24)		0.47	74.76 (9.75)		0.01	75.15 (10.84)		0.12
	MT	79.26 (10.00)		79.23 (9.68)		0.95	77.90 (9.13)		0.17	77.62 (11.76)		0.23	78.29 (8.87)		0.35
TBR (< 54 mg/dL)	UC	0.57 (0.94)		0.54 (0.78)		0.84	0.66 (1.01)		0.55	0.47 (0.82)		0.75	0.39 (0.61)		0.70
	MT	0.47 (0.81)		0.55 (1.21)		0.39	0.59 (0.76)		0.11	0.67 (0.96)		0.05	0.46 (0.73)		0.75
Time of use of automatic mode	UC	72.41 (33.18)		87.55 (15.55)		0.03	88.53 (14.42)		0.04	88.28 (13.84)		0.04	84.81 (16.91)		0.30
	MT	84.90 (19.14)		85.36 (24.56)		0.87	88.00 (19.15)		0.20	85.28 (21.15)		0.76	85.95 (17.78)		0.59
*Basal treatment*															
TIR (70–180 mg/dL)	MDI	76.93 (10.73)		77.79 (9.46)		0.38	76.37 (9.45)		0.39	76.97 (9.20)		0.29	76.36 (10.16)		0.20
	SAP	80.66 (8.95)		79.52 (9.32)		0.14	79.19 (9.30)		0.16	76.52 (13.65)		0.07	78.67 (8.63)		0.24
TBR (< 54 mg/dL)	MDI	0.58 (0.93)		0.65 (1.30)		0.59	0.72 (0.92)		0.18	0.51 (0.82)		0.67	0.68 (1.07)		0.55
	SAP	0.37 (0.72)		0.41 (1.01)		0.72	0.47 (0.70)		0.39	0.35 (0.48)		0.47	0.51 (0.68)		0.03
Time of use of automatic mode	MDI	79.20 (27.18)		87.32 (20.46)		0.05	88.55 (15.33)		0.02	87.01 (26.85)		0.05	87.57 (15.70)		0.07
	SAP	84.76 (19.25)		84.17 (25.00)		0.87	87.65 (20.79)		0.42	85.00 (19.60)		0.98	83.40 (19.24)		0.54

Data presented as mean and standard deviation. TIR: time in range; TAR: time above range; TBR: time below range; GMI: glucose management indicator; CV%: coefficient of variation; MT. Multidisciplinary team; UC: Usual care; MDI: multiple daily injection; SAP: Sensor Augmented Pump with Low Glucose Suspend or Sensor Augmented Pump Therapy with Predictive low-glucose management. *P* value of paired *t* test comparing with baseline values

No significant changes were found in TBR < 70 mg/dL, < 54 mg/dL or in glycemic variability throughout the follow-up (Table 2). This finding was maintained when analyzing according to the follow-up modality. However, a statistically significant increase in TBR < 54 mg/dl was found in the subgroup previously treated with SAP at 12 months of follow-up (0.51 ± 0.68, *p* = 0.03) (Table 3). No ketoacidosis or SH events were reported.

### Adherence

All patients showed sensor use and adherence to AM more than 80% of the time from baseline to 12-month follow-up. A statistically significant increase in adherence to AM was found in the UC subgroup at three (*p* = 0.03), six (*p* = 0.03) and nine months (*p* = 0.04) (Table 3). These changes were not evident in MT as they had a higher basal adherence to auto mode. At the end of follow-up, adherence to AM remained above 80% regardless of the initial treatment (Table 3). None of the patients discontinued automatic mode at study closure.

## Discussion

The use of the HCL systems allows to achieve TIR (70–180 mg/dL) goals early and persistently during long-term follow-up in a population with T1D at high risk of hypoglycemia with a reduction in exposure to hyper and hypoglycemia and low glycemic variability. Previous therapy and follow-up team did not affect the glycemic control or adherence in our study. In our study, the mean basal TIR (70–180 mg/dl) measured two weeks after starting AM use was 78.6 ± 9.9%, and it remained at similar levels until the end of follow-up.

Berget et al. compared the glycemic control between young vs adult population. They found a higher TIR in adults and older adults compared with youth and young adults, which did not change across time (70.0 ± 1.2% and 75.1 ± 2.2% at month 12, respectively; *p* = 0.60 for both groups) [8]. Similar to our data, both TIR during follow-up and the use of auto mode were above 80% [8]. Other previous real-world studies have shown an increase in TIR after 1-year of follow-up [10, 11]. However, some studies still report TIR below 70% [10, 12]. A statistically significant increase in TBR < 54 mg/dl was found in the subgroup previously treated with SAP; however, it was not clinically relevant and both groups remained below 1%. These findings are similar to other studies performed in a real-world setting [11] and reinforces the benefit in patients followed using telemedicine. It is important to note that there were no severe hypoglycemic (SH) events after the start of HCL.

The use of auto mode more than 70% of the time correlates well with improved glycemic control [8, 10]. However, “real world” studies have reported a dropout rate of auto mode close to 30% in adult [10, 11] and pediatric [10] populations, and a drop in the time of use of the automatic mode with the passage of time [10]. The main reasons for auto mode discontinuation included problems related with the sensor and the supplies, fear of hypoglycemia, and incompatibility with the practice of sports [10]. Additionally, low adherence to auto mode use has been associated with young population [12]. In our study, none of the patients discontinued the auto mode, and they used it more than 80% of time, from the start of the study and throughout the follow-up. This high adherence could be related to the inclusion of adult population with sensor compliance more than 90% of the time. Likewise, to 100% coverage of HCL therapy by the Colombian health system. The subgroup of patients who continued their follow-up through a multidisciplinary program achieved a TIR close to 80% after 1-year of follow-up. Even though it was not statistically significant, these patients achieved higher TIR, lower TAR, TBR and CV% compared with the patients under usual care. These data suggests that it is necessary to design larger clinical trials that allow a more careful evaluation of the impact of multidisciplinary follow-up versus usual care.

The use of telemedicine has increased after 2020 related to COVID-19 pandemic [13], its advantages are regular consultation and improved connectivity, which could explain the significant increase in TIR by 5% [14] and reduction in A1c in some studies [15]. Therefore, it was necessary to develop a multidisciplinary program through virtual platforms for users of HCL systems that would comply with government regulations and allows frequent follow-up [6]. The population included in this study had a virtual training program with which they achieved a significant increase of TIR from 77.3% ± 11.3 in manual mode to 81.6 ± 76% (*p* < 0.001) using auto mode, for this reason they were familiarized with telemedicine modality and its advantages [6]. Also, telemonitoring of glucose metrics has been widely used during the pandemic in people with diabetes [14]. Similar to our findings, different cohorts have shown maintenance or even improvement in glycemic control, suggesting that glucose control was not affected despite discontinuation. of face-to-face care in clinical diabetes services[14].

To our knowledge, this is the largest prospective “real world” study including high risk hypoglycemia population with long term follow-up through telemedicine. This study reinforces the importance of adherence to sensor use and auto mode to achieve higher TIR.

Among the limitations, this is an observational study without a control group, so we cannot compare patients with or without HCL treatment. However, our data demonstrates that the use of telemedicine is a good alternative to standard of care.

## Conclusion

The use of the HCL system makes it possible to achieve TIR goals (70–180 mg/dL) safely, early and sustained up to 1 year of follow-up in patients with T1D and high risk of hypoglycemia followed through telemedicine. Prior therapy and follow-up modality did not affect glycemic control or adherence in our study. Our data suggests that follow-up by a multidisciplinary team achieves better TIR, TAR, TBR and %CV than the subgroup of patients under usual care. However, it is necessary to design larger clinical trials that allow evaluating the impact of multidisciplinary follow-up vs usual care.

## Abbreviations

A1c: Glycosylated hemoglobin
AM: Auto mode
CV%: Coefficient of variation
CGM: Continuous glucose monitoring
GMI: Glucose management indicator
PLGM: Predictive low-glucose management
SAP: Sensor-augmented pump therapy
T1D: Type 1 diabetes
TAR: Time above the range
TBR: Time below the range
TIR: Time in range
HCL: Hybrid closed Loop
AID: Automated insulin delivery system
MDI: Multiple doses of Insulin
SH: Severe hypoglycemia
UH: Hypoglycemia unawareness
